# Efficacy and safety of mycophenolate mofetil therapy in neuromyelitis optica spectrum disorders: a systematic review and meta-analysis

**DOI:** 10.1038/s41598-020-73882-8

**Published:** 2020-10-07

**Authors:** Sakdipat Songwisit, Punchika Kosiyakul, Jiraporn Jitprapaikulsan, Naraporn Prayoonwiwat, Patompong Ungprasert, Sasitorn Siritho

**Affiliations:** 1 Department of Medicine, Faculty of Medicine Siriraj Hospital, Mahidol University, Bangkok, Thailand; 2 Division of Neurology, Department of Medicine, Faculty of Medicine Siriraj Hospital, Mahidol University, 2 Wanglang Road, Bangkok Noi, Bangkok, 10700 Thailand; 3 Siriraj Neuroimmunology Center, Faculty of Medicine Siriraj Hospital, Mahidol University, Bangkok, Thailand; 4 Department of Rheumatic and Immunologic Diseases, Cleveland Clinic, Cleveland, OH USA; 5 Bumrungrad International Hospital, Bangkok, Thailand

**Keywords:** Immunology, Neurology

## Abstract

Mycophenolate mofetil (MMF) is an immunosuppressive agent (IS) which is widely prescribed in neuromyelitis optica spectrum disorder (NMOSD) patients. We aim to assess the efficacy and safety of MMF in controlling relapse and disease severity. Eligible studies obtained from the EMBASE and Ovid MEDLINE databases were studies of NMOSD patients treated with MMF, which reported treatment outcomes as Annualized Relapse Rate (ARR) or Expanded Disability Status Scale (EDSS) before and after treatment. Fifteen studies included 1047 patients, of whom 915 (87.4%) were aquaporin-4 immunoglobulin seropositive. The total number of patients that received MMF was 799. A meta-analysis on ARR was conducted in 200 patients from 4 studies and on EDSS in 158 patients from 3 studies. The result showed a significant improvement with a mean reduction of 1.13 [95% confidence interval (CI) 0.60–1.65] in ARR, and a mean reduction of 0.85 (95% CI 0.36–1.34) in EDSS after MMF therapy. Adverse events occurred in 106 (17.8%) of 594 patients during MMF therapy. This systematic review and meta-analysis showed that using MMF as a preventive therapy in NMOSD patients can significantly reduce relapse rates and improve disease severity with acceptable tolerability.

## Introduction

Neuromyelitis optica spectrum disorder (NMOSD) is an immune-mediated inflammatory disease of the central nervous system with aquaporin-4 immunoglobulin G (AQP-4 IgG) as a pathogenic autoantibody. The original notion that NMOSD is a monophasic attack consisting of optic neuritis (ON) and transverse myelitis (TM) was later replaced by recurrent courses in most cases^1,2^. Unlike multiple sclerosis (MS), disability in NMOSD patients correlates with the number of recurrent attacks rather than disease progression. Therefore, most treatments aim to prevent relapses^3^.

Although there are several newly approved medications in randomization studies such as eculizumab, satralizumab, and inebilizumab for maintenance treatment in NMOSD, long term benefits, and side effects need evaluation^4–7^. Other immunosuppressive agents (IS), including corticosteroids, azathioprine (AZA), mycophenolate mofetil (MMF), methotrexate, cyclophosphamide (CYP), mitoxantrone, and rituximab (RTX) have been used for decades and still remain the standard initial treatment for attack prevention in patients with NMOSD^8,9^.

Among those, MMF, a prodrug of mycophenolic acid (MPA), is a reversible, non-competitive inhibitor of inosine-5′-monophosphate dehydrogenase (IMPDH). MPA depletes guanosine nucleotides preferentially in T and B lymphocytes and inhibits their proliferation. Therefore, treatment with MMF suppresses both cell-mediated immune responses and antibody formation^10^.

Application of MMF has extended from post organ transplantation to many autoimmune diseases, including NMOSD. The first case series that suggested using MMF as a preventive treatment for relapses in NMOSD patients was published in 2009^11^. Subsequent studies have also corroborated the same benefit of MMF as a preventive therapy in NMOSD and its ability to reduce neurological impairment.

Randomized controlled trials of MMF in NMOSD patients are not available, as there are only case series, mostly with a retrospective study design. Therefore, we conducted a systemic review to evaluate the efficacy and adverse drug reactions (ADRs) of MMF in NMOSD patients.

## Materials and methods

### Study selection

Two investigators (S.S. and P.K.) independently searched for eligible published peer-reviewed studies indexed in Ovid MEDLINE and EMBASE databases from inception to April 2020. The search terms “neuromyelitis optica spectrum disorder” and “mycophenolate mofetil” were used (supplementary data), and was limited to English-language human studies. Eligible studies could be either randomized-controlled trials or cohort studies/case series that investigated MMF's efficacy in NMOSD patients. Changes in either the annualized relapse rate (ARR) ratio or Expanded Disability Status Scale (EDSS) score before and after treatment must be reported. To avoid the non-representativeness of cases, case series that included fewer than 3 patients were excluded. The studies were reviewed in full-length to assess the appropriateness for their inclusion in the systematic review. Any differences in the determination of study eligibility between the two investigators mentioned above were re-evaluated, and the disagreement was resolved by discussion with other investigators (J.J. and P.U.).

### Data extraction

The extracted data included year and country of publication, study design, diagnostic criteria of NMO/NMOSD, demographic data of patients (patient population, female sex ratio, age of onset, follow-up duration, mean disease duration, ARR, EDSS score before and after MMF treatment, and AQP4-IgG serostatus), MMF treatment protocols (dose and duration of MMF treatment, and previous or concurrent therapy) and outcome measures.

### Efficacy and safety measures

For the primary outcome on efficacy, differences in ARR, and the mean and median EDSS scores before and after MMF treatment were assessed. Safety outcomes included the proportion of deaths, drug withdrawals due to toxic effects, and the ADRs related to MMF use such as infection, malignancy, and abnormal laboratory results if available. Detail of adverse events in 15 studies on mycophenolate mofetil in NMOSD was displayed in Table 3.

### Quality assessment and statistical analysis

Quality assessment for the included observational studies was performed using the Newcastle–Ottawa quality assessment scale, which consists of 3 domains: (a) selection of the participants; (b) comparability between the groups; and (c) ascertainment of the outcome in cohort studies^12^. Differences in the assessment were discussed and resolved with consensus among investigators.

Continuous and dichotomous data were both included in this study. Continuous data (ARR and EDSS) were reported as a median with range or mean with standard deviation (SD) depending on available data in the original articles. A number presented dichotomous data (i.e., number of ADRs, number of AQP4-IgG seropositive patients) with a percentage.

Meta-analysis was performed using Review Manager 5.3 software from the Cochrane Collaboration (London, United Kingdom). Mean pre- and post-treatment ARR, as well as mean pre- and post-treatment EDSS along with their SD, were extracted from each study, and the mean difference (MD) was calculated. If the study reported a 95% confidence interval (95% CI) instead of SD, the SD would be calculated from an upper limit of the 95% CI. Statistical heterogeneity was evaluated using Cochrane's Q test and was complemented with the I^2^ statistic, which quantifies the proportion of the total variation across studies due to heterogeneity rather than chance. An I^2^ value from 0 to 25% represents insignificant heterogeneity, 26–50% represents low heterogeneity, 51–75% represents moderate heterogeneity, and > 75% represents high heterogeneity^13^. A result is considered statistically significant if a 95% CI of the MD did not include a null value; zero, for continuous data.

### Consent for publication

I, the corresponding author, give my consent for information about the manuscript to be published in Scientifics Reports.

## Results

### Study identification and selection

**Figure 1:**
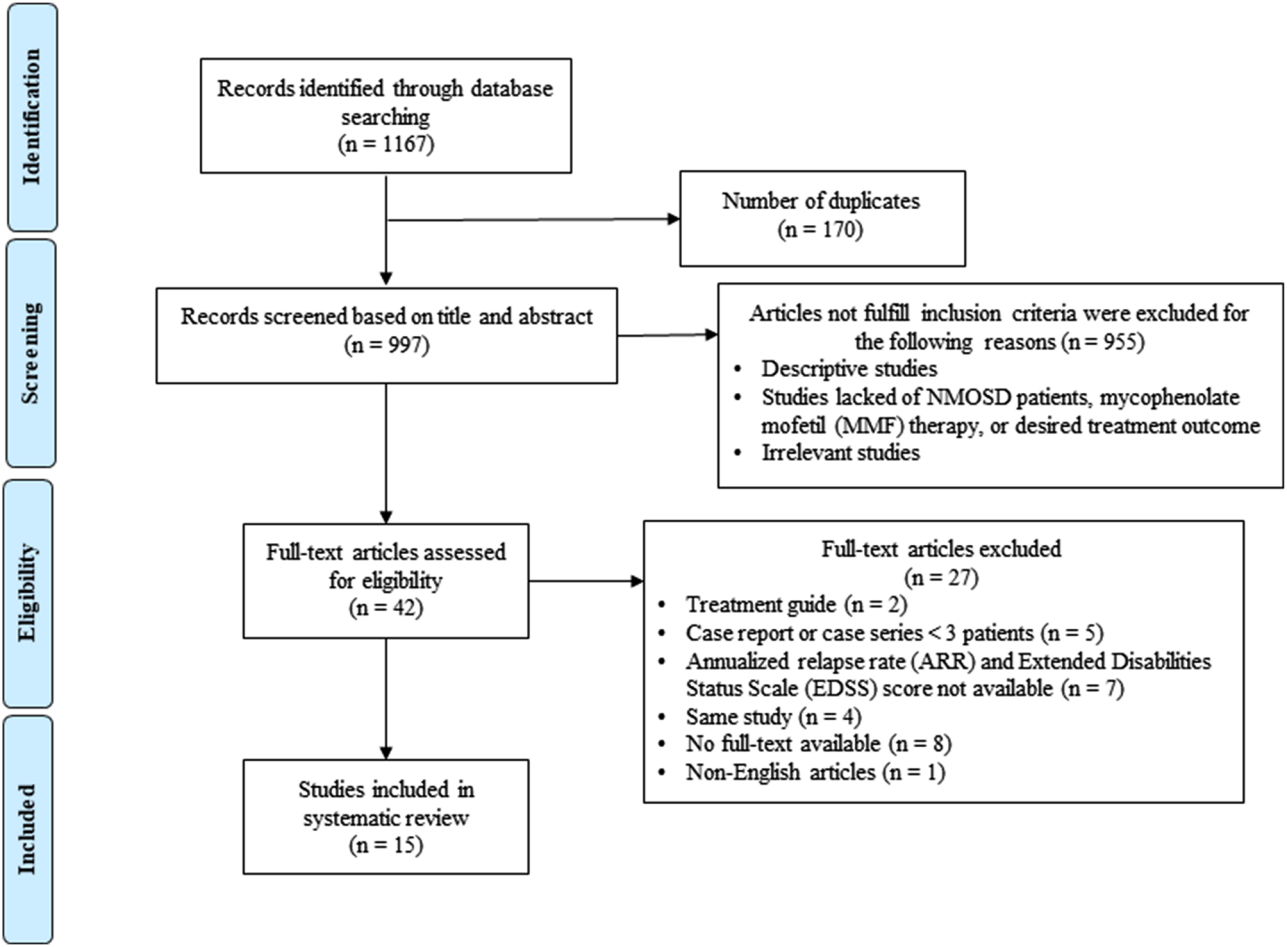
The PRISMA flow diagram of this systematic review.

There were 1167 articles identified through database searching from Ovid MEDLINE and EMBASE. After excluding 170 duplicates, a total of 997 studies were screened by titles and abstracts. Forty-two studies were analyzed for eligibility assessment. Of these 42 studies, 27 studies were excluded (2 studies were reviews, 5 studies were case reports or case series with fewer than three patients, 7 studies had no reported ARR or EDSS pre- and post-treatment, 4 studies were duplicates, 8 studies had no full-text available, and 1 study had no English article available). As a result, 15 studies (10 retrospective and 5 prospective) published during 2009–2020 met our study criteria and were included in the systemic analysis (Fig. 1). None were randomized controlled trials, 14 were cohort studies, and 1 was a case series.

### Assessment of risk of bias

**Figure 2:**
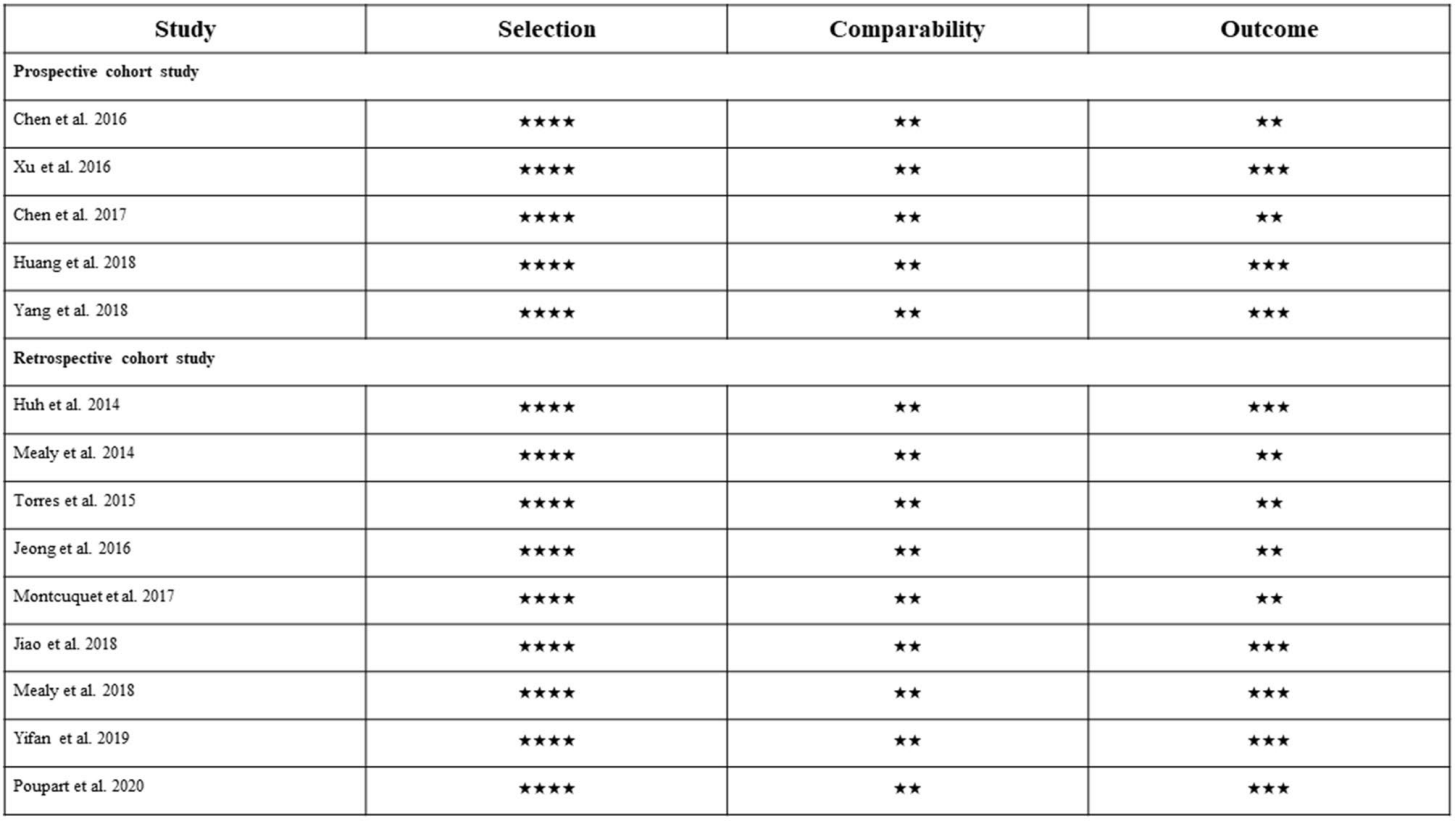
The quality assessment of 14 observational studies by using Newcastle–Ottawa Scale.

Figure 2 shows the result of the 14 cohort studies' quality assessment by using the Newcastle–Ottawa Quality Assessment scale. All observational studies were excellent in methodological quality with a total of 8 out of 9 stars in 6 studies and 9 out of 9 stars in 8 studies.

### Demographic and clinical characteristics

The characteristics and demographic data for the 15 studies are described in Table 1 and the supplementary table. There were a total of 1047 patients (799 of them were treated with MMF). The total number of female patients was 915 (overall 87.4%), with the female proportion varying from 73.8% to 93.3% for each individual study. The AQP4-IgG serostatus was reported for all patients, of whom 886 patients (84.6%) were AQP4-IgG seropositive. Diagnosis of NMO/NMOSD was given according to the 2006^2^, 2007^14^, or 2015^1^ criteria.

**Table 1:** Baseline characteristics of 15 studies in neuromyelitis optica spectrum disorders treated with mycophenolate mofetil.

References	Study design	Diagnosis of NMO/ NMOSD	Number with positive AQP4 antibody/total number (%)	Number of females/total number (%)	Number of patients treated with MMF	Age of onset, years old	Dose of MMF	Other immune-suppressive (IS) therapy prior to MMF; number of patients (%)	Concurrent use of corticosteroid; number of patients (%)	Assessment of treatment response and duration of follow-up/treatment
Jacob et al.^11^	Retrospective case series	The 2006 NMO criteria OR	23/24 (95.8%)	19/24 (79.2%)	24	Median 56 (range 34–77)	Median 2000 mg/day (range 750–3000)	17 (70.8%)	9 (37.5%)	Assessment: at final follow-up visit
		The 2007 NMOSD criteria								Duration of follow-up: median 28 months (range 18–89 months)
Huh et al.^27^	Retrospective cohort	The 2006 NMO criteria OR	52/58 (89.7%)	50/58 (86.2%)	58	Median 34 (range 10–53)	1000–2000 mg/day	22 (37.9%)	1 (1.72%)	Assessment: at latest MMF treatment
		The 2007 NMOSD criteria								Duration of treatment: median 20 months (range 4–67 months)
Mealy et al.^32^	Retrospective cohort	The 2006 NMO criteria OR	17/28 (60.7%)	26/28 (92.9%)	28	Median 36 (range 19–74)	1000–2000 mg/day	8 (28.6%)	13 (46.4%)	Duration of treatment: median 26 months (range 6–68 months)
		The 2007 NMOSD criteria								
Torres et al.^23^	Retrospective cohort	The 2006 NMO criteria OR	4/11 (36.4%)	10/11 (90.9%)	11	Median 37 (range 18–68)	NR	7 (63.6%)	NR	Duration of follow-up: median 23 months (range 13–60 months)
		The 2007 NMOSD criteria								
Chen et al.^16^	Prospective cohort	The 2006 NMO criteria OR	52/62 (83.9%)	58/62 (93.5%)	62	Mean 38.7 (SD 12.0)	20 mg/kg	7 (11.3%)	24 (38.7%)	Assessment: at final follow-up visit
		The 2007 NMOSD criteria								Duration of follow-up: median 18 months (range 6–49 months)
Jeong et al.^18^	Retrospective cohort	The 2006 NMO criteria OR	32/34 (94.1%)	29/34 (85.3%)	34	Median 35 (range 10–53)	1500–2000 mg/day	None (0%)	9 (26.4%)	Duration of treatment: median 26.1 months (range 5.5–68.6 months)
		The 2007 NMOSD criteria								
Xu et al.^19^	Prospective cohort	The 2015 IPND	33/38 (86.8%)	32/38 (84.2%)	38	Mean 28.7 (SD 13.0)	1500 mg/day	None (0%)	All (100%)	Duration of treatment: median 15.2 months (range 6.6–26.4 months)
Chen et al.^17^	Prospective cohort	The 2006 NMO criteria OR	89/105 (84.8%)	97/105 (92.4%)	105	Mean 44.0 (SD 12.1)	20 mg/kg/d	None (0%)	49 (46.6%)	Assessment: at final follow-up visit
		The 2007 NMOSD criteria								Duration of treatment: median 17 months (range 6–78 months)
Montcuquet et al.^20^	Retrospective Cohort	The 2015 IPND	45/67 (67.2%)	50/67 (74.6%)	67	Median 37.9 (range 6–67)	2000 mg/day	None (0%)	16 (23.9%)	Duration of treatment: median 24 months (range 1–156 months)
Huang et al.^25^	Prospective cohort	The 2006 NMO criteria OR	90/90 (100%)	84/90 (93.3%)	90	Median 36 (range 10–65)	1000 mg/day	20 (22.2%)	All (100%)	Duration of follow-up: median 13.5 months
		The 2015 IPND								
Jiao et al.^15^	Retrospective cohort	The 2006 NMO criteria OR	74/86 (86.0%)	77/86 (89.5%)	86	Median 43 (range 6–68)	High dose (1750–2000 mg)	56 (65.1%)	65 (76%)	Assessment: at final follow-up visit
		The 2007 NMOSD criteria					Moderate dose (1250–1500 mg)			Duration of treatment: median 20 months (range 6–89 months)
							Low dose (≤ 1000 mg)			
Mealy et al.^24^	Retrospective cohort	The 2015 IPND	208/245 (84.9%)	216/245 (88.2%)	103	Median 37 (range 7–79)	1500–2000 mg/day	Some had glatiramer acetate	None (0%)	Duration of treatment: median 36 months (range 6–92 months)
										Duration of follow-up: median 95 months (mean, 103 months)
Yang et al.^21^	Prospective cohort	The 2015 IPND	13/30 (43.3%)	26/30 (86.7%)	30	Mean 42.6 (SD 11.7)	1000 mg/day	None (0%)	28 (93.3%)	Assessment: at final follow-up visit
										Duration of follow-up: median 28.5 months (range 19–42 months)
Zhou et al.^26^	Retrospective cohort	The 2006 NMO criteria OR	Pediatric group: 23/31 (74.2%)	Pediatric group: 25/31 (80.6%)	4	Pediatric group: Median 14 (range 10–17)	1000 mg/day	Some had AZA or CYP	All (100%)	Assessment: at final follow-up visit
		The 2015 IPND	Adult group: 96/96 (100%)	Adult group: 85/96 (88.8%)	17	Adult group: Median 35 (range 18–96)	1000 mg/day		All (100%)	Duration of follow-up: median 17 months (range 8–26 months)
Poupart et al.^22^	Retrospective cohort	The 2015 IPND	35/42 (83.3%)	31/42 (73.8%)	42	Mean 41.4 (SD 17.6)	1000–2000 mg/day	None (0%)	8 (19.1%)	Median 35 months (interquartile range 3.2)

*AQP4* Aquaporin4, *AZA* azathioprine, *CYP* cyclophosphamide, *IS* immunosuppressive, *kg* kilogram, *mg* milligram, *MMF* mycophenolate mofetil, *NMO* neuromyelitis optica, *NMOSD* neuromyelitis optica spectrum disorders, *NR* not reported, *ON* optic neuritis, *SD* standard deviation, *IPND* International Panel for Neuromyelitis optica Diagnosis.

### Treatment regimens

MMF was administered at a dose of 1000–2000 mg/day in most of the studies. One study by Jiao et al. reported categorized dosages (1000 mg/day or less, 1250–1500 mg/day, 1750–2000 mg/day as low, moderate, and high dose, respectively)^15^. Studies by Chen et al. prescribed a 20 mg/kg dosage^16,17^. Of the 799 patients treated with MMF, MMF was used as a first-line therapy in 6 studies^17–22^ with a total of 316 patients (39.5%). The other 9 studies included patients who had suboptimal treatment from prior IS, including AZA, CYP, mitoxantrone, fingolimod, hydroxychloroquine sulfate, beta-interferons, and glatiramer acetate; however, the studies did not contain detailed information of the proportionate use. Data on concomitant corticosteroids were available in all but one study^23^. The proportion of steroid use ranged from 0^24^ to 100%^19,25,26^. In four studies^19,21,25,26^, 177 patients (22.2%) had been taking oral corticosteroid at the time of MMF treatment. Two studies^24,27^ were conducted with 160 patients (20.0%) who did not receive corticosteroid during MMF treatment. The remaining 10 studies contained both groups of patients.

### Treatment outcome analysis

The efficacy of MMF treatment determined by changes in ARR and EDSS is shown in Table 2. The median follow-up duration ranged from 13.5^25^ to 95 months^24^, with less than 24 months in 8 studies and 24 months or more in 7 studies (Table 1). All studies gave neither detail of the MRI findings nor evaluated it as a treatment outcome.

**Table 2:** Changes in Expanded Disabilities Status Score and annualized relapse rate after treatment with mycophenolate mofetil.

Author	EDSS			Improved or stabilized EDSS (%)	ARR			Relapse free rate (%)
	Median pre-treatment (range)	Median post-treatment (range)	*P* value		median pre-treatment (range)	Median post-treatment (range)	*P* value	
Jacob et al.^11^	6.0 (0.0–8.0)	5.5 (0.0–10)	0.17	91	1.15 (0.23–11.78)	0.18 (0.00–1.50)	< 0.01	46
Huh et al.^27^	3.0 (0.0–8.0)	2.5 (0.0–7.0)	0.01	91	1.50 (0.30–11.80)	0.00 (0.00–2.60)	< 0.01	60
	3.2 (2.2)^a^	2.7 (1.9)^a^			2.6 (2.7)^a^	0.5 (0.8)^a^		
Mealy et al.^32^	NR	NR	NR	NR	2.61 (NR)	0.33 (NR)	< 0.01	64
Torres et al.^23^	4.0 (3.0–6.5)	5.0 (NR)	0.46	NR	1.06 (0.84–2.31)	0.39 (NR)	< 0.05	27
Chen et al.^16^	4.0 (0.5–8.0)	2.0 (0.5–7.5)	< 0.01	95.2	1.20 (0.20–7.00)	0.00 (0.00–1.70)	< 0.01	58.1
	4.1 (2)^a^	2.8 (2.1)^a^			1.7 (1.2)^a^	0.4 (0.5)^a^		
Jeong et al.^18^	3.0 (0.0–7.0)	2.0 (0.0–7.0)	< 0.01	NR	1.54^c^	0.18^c^	< 0.01	64.7
Xu et al.^19^	2.0 (0.0–9.0)	2.0 (0.0–8.5)	< 0.01	97.4	0.80 (0.00–8.00)	0.00 (0.00–1.40)	0.05	NR
	2.7 (2)^a^	2.0 (1.8)^a^			1.0 (1.0)^a^	0.1 (0.3)^a^		
Chen et al.^17^	3.0 (0.5–8.0)	2.0 (0.5–7.5)	< 0.01	NR	1.20 (0.10–7.00)	0.00 (0.00–2.00)	< 0.01	56.2
**Montcuquet et al.**^20^								
Total	4.0 (0.0–8.5)	3.8 (0.0–10.0)	< 0.05	NR	1.00 (0.10–3.20)	0.00 (0.00–3.00)	< 0.05	49.3
AQP4-pos	4 (0–8.0)	4 (0–8.5)	NR	NR	1 (0.17–3.0)	0.21 (0–1.12)	NR	46.7
AQP4-neg	3.5 (0–8.5)	4 (0–10)	NR	NR	0.9 (0.1–3.2)	0 (0–0.8)	NR	61.3
Huang et al.^25^	4.0 (0.0–8.5)	3.0 (0.0–8.5)	< 0.01	90	1.02 (0.00–19.21)	0.00 (0.00–2.44)	< 0.01	73
Jiao et al.^15^	3.0 (0.0–8.5)	2.5 (0.0–8.5)	0.01	87	1.40 (0.10–11.00)	0.00 (0.00–2.80)	< 0.01	64
**Mealy et al.**^24^								
Total	NR	NR	NR	NR	NR	NR	NR	NR
AQP4-pos	NR	NR	NR	NR	1.79	0.29	< 0.01	64.7
AQP4-neg	NR	NR	NR	NR	1.45	0.30	< 0.01	77.8
Yang et al.^21^	3.5 (2.0–8.5)	2.0 (0.5–7.0)	< 0.01	100	0.90 (0.00–5.00)	0.00 (0.00–2.40)	< 0.01	60
Zhou et al.^26^	NR	NR	NR	NR	1.00 (0.23—3.43) in adult patients	0.00 (0.00—0.71) in adult patients	< 0.01	80% in adult patients
					0.98 (0.35–2.11) in pediatric patients	0.28 (0–0.71) in pediatric patients		50% in pediatric patients
Poupart et al.^22^	NR	NR	NR	NR	0.71 (0.43–1.15)^b^	0.20 (0.11–0.35)^b^	NR	NR

*EDSS* Expanded Disability Status Scale, *ARR* annual relapse rate, *NR* not reported.

^a^Mean (SD).

^b^Mean (95% CI).

^c^ARR as number of relapses by person-year.

Since, there were only 4 studies^16,19,22,27^ with ARR and 3 studies^16,19,27^ with EDSS that reported the mean and standard deviation (SD), they were included in the meta-analysis. The remaining 8 studies did not report the mean and SD; therefore, they could not be statistically analyzed and were omitted from the meta-analysis. Nevertheless, we did a qualitative analysis of the 15 studies, which were displayed in Table 2.

### Qualitative analysis

All 15 studies reported the median ARR before and after treatment. One study^22^ reported ARR by using “the total number of relapses per patient-year” while the remaining studies defined ARR as the number of relapses per year. All but one study^22^ demonstrated significant ARR reduction after receiving MMF (*p* < 0.05). The relapse-free rate was 60% (ranged from 27 to 80%).

For the 11 studies reporting EDSS as a treatment outcome, 7 studies revealed stabilization or improvement of disability in patients receiving MMF treatment measured by EDSS with the proportion varying from 87 to 100%. Eleven studies reported median EDSS before and after MMF treatment. Of those, 9 showed significant post-treatment median EDSS improvement (*p* < 0.05).

There were 2 studies^20,24^ that categorized patients into seronegative and seropositive NMOSD groups and reported efficacy on MMF treatment separately in each group. Montcuquet et al. showed a reduction in the median pre-treatment ARR from 1 to post-treatment ARR of 0.21 in seropositive NMOSD and 0.9–0 in seronegative NMOSD and a relapse-free rate of 46.7% and 61.3%, respectively. However, no changes in EDSS was demonstrated^20^. The other study also revealed the reduction in the median pre- and post-treatment ARR of 1.79–0.29 in seropositive NMOSD and 1.45–0.30 in seronegative NMOSD, and a relapse-free rate of 64% and 77.8%, respectively^24^ (Table 2).

### Meta-analysis: efficacy of the reduction of ARR

**Figure 3:**
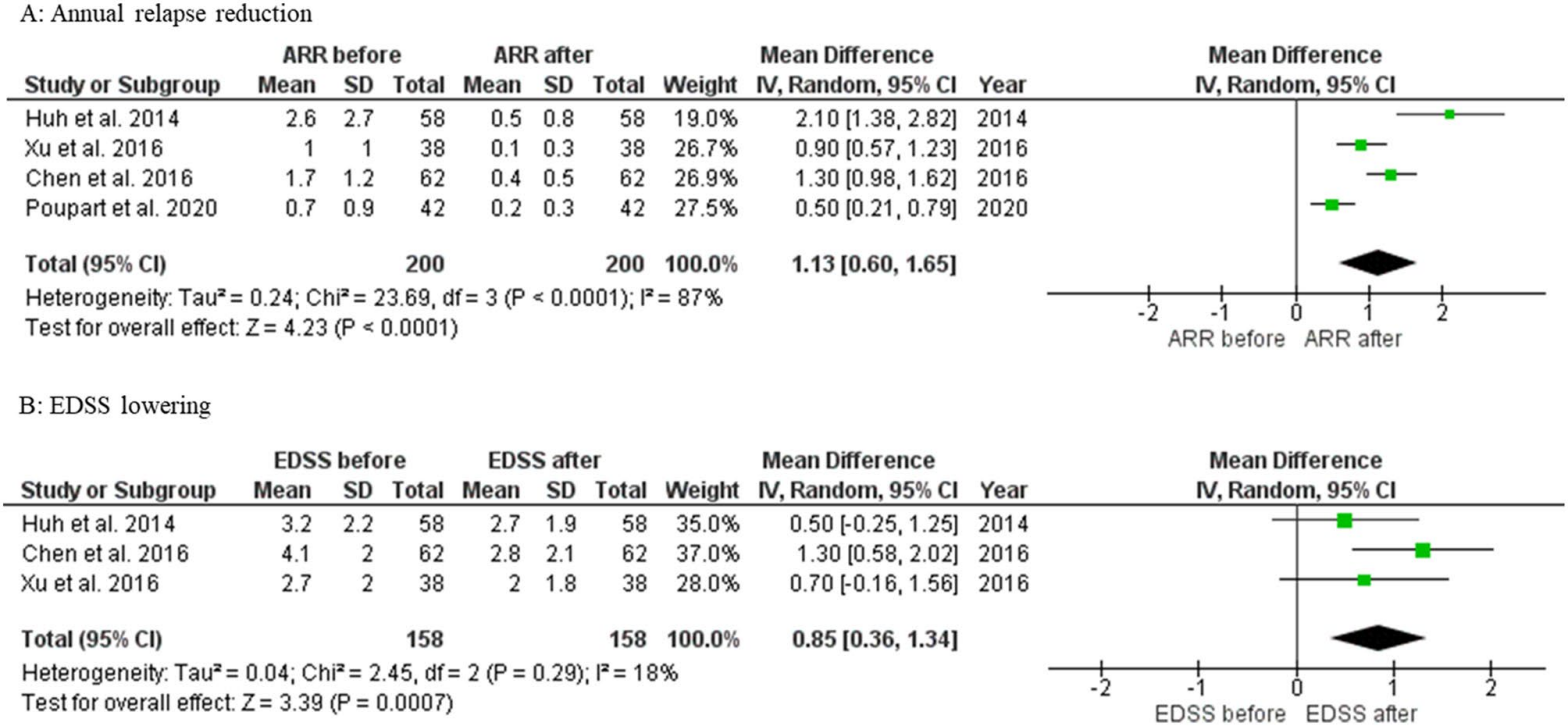
Meta-analysis on efficacy of MMF in annual relapse reduction and EDSS lowering.

All 4 studies^16,19,22,27^, including 200 NMOSD patients with the majority of patients being AQP4-positive (80–90%), showed a significant ARR reduction with the mean reduction of 1.13 (95% CI 0.60–1.65) after MMF therapy (1000–2000 mg/day for 15.2–35 months), compared to the ARR at the initiation of treatment. (Fig. 3a).

### Meta-analysis: efficacy on the EDSS

All 3 studies^16,19,27^ with 158 NMOSD patients showed a significant reduction in EDSS after MMF therapy with a mean reduction of 0.85 (95% CI 0.36–1.34). Moreover, the Chen study^16^ showed a large decrease in disability measured by EDSS from baseline of moderately severe (EDSS 4.1) to full independence (EDSS 2.1). (Fig. 3b).

### Safety

ADRs are summarized in Table 3. Of 799 patients, data on the safety of MMF therapy were recorded for 594 patients. One hundred and six patients (17.8%) were reported to have ADRs. One of the most common ADRs were infections (33 patients; 5.6%)—including respiratory infection/pneumonia (12 patients; 2.0%), urinary tract infection (8 patients; 1.3%), herpes zoster infection (8 patients; 1.3%), herpes simplex infection (2 patients; 0.3%), and abnormal liver function tests (27 patients; 4.5%). The other common ADRs were hair loss (17 patients; 2.9%), gastrointestinal (GI) side effects (14 patients; 2.4%)—including nausea, diarrhea /abdominal pain, and constipation—bone marrow suppression (16 patients; 2.7%)—including anemia (6 patients; 1.0%), agranulocytosis (1 patient; 0.2%), leukopenia (8 patients; 1.3%), thrombocytopenia (1 patient; 0.2%), and amenorrhea in 3 patients (0.5%). Uncommon documented side effects were headaches, phlegm on normal CT chest, chronic dermopathy of the hand, rectal cancer, renal insufficiency, rash, hypotension, fatigue, easy bruising, anxiety, and sun sensitivity. Data on the discontinuation of MMF was available for 687 patients. Twenty-seven patients (3.9%) discontinued MMF due to ADRs such as rash, agranulocytosis, leukopenia, thrombocytopenia, arthromyalgia, GI side effects, and amenorrhea. All ADRs were reversible after discontinuation of MMF. One patient was discovered to have high serum CEA (carcinogenicembryonic antigen). Three patients died during the MMF treatment: one with EDSS 8.5 succumbed from immobilization complications, another developed disseminated varicella-zoster with acute respiratory distress syndrome, and the other had—according to death certificate documents—“cardiopulmonary failure; respiratory drive failure and Devic’s disease”^11,20,25^.

**Table 3:** Adverse events in 15 studies on mycophenolate mofetil in neuromyelitis optica spectrum disorders.

Author	Total number of patients	Number of patients with adverse events (%)	Adverse events	Number of events (%)	Total number of discontinuation due to adverse effects (%)
Jacob et al. ^11^	24	6 (25%)	Headache	1 (4.2%)	1 (4.2%) due to low white blood cell counts
			Constipation	1 (4.2%)	
			Easy bruising	1 (4.2%)	
			Anxiety	1 (4.2%)	
			Hair loss	1 (4.2%)	
			Diarrhea and abdominal pain	1 (4.2%)	
			Low white blood cell counts	1 (4.2%)	
Huh et al.^27^	58	14 (24.13%)	Rash	1 (1.7%)	1 (1.7%) due to rash
			Amenorrhea	2 (3.4%)	
			Herpes zoster	1 (1.7%)	
			Cystitis	3 (5.2%)	
			Pneumonia	1 (1.7%)	
			Hypotension	1 (1.7%)	
			Fatigue	1 (1.7%)	
			Mild hair loss	4 (6.9%)	
Mealy et al.^32^	28	NR	NR	NR	0 (0%)
Torres et al.^23^	11	4 (36%)	Sun sensitivity	NR	NR
			Recurrent infection	NR	
Chen et al.^16^	62	3 (4.8%)	Mild hair loss	2 (3.2%)	0 (0%)
			Mildly elevated liver enzyme (After reused, no elevated liver enzyme)	1 (1.6%)	
Jeong et al.^18^	34	NR	NR	NR	0 (0%)
Xu et al.^19^	38	2 (5.3%)	Agranulocytosis	1 (2.6%)	2 (5.3%) due to agranulocytosis, amenorrhea
			Amenorrhea	1 (2.6%)	
Chen et al.^17^	105	5 (4.8%)	Mild hair loss	3 (2.9%)	0 (0%)
			Mildly elevated liver enzyme	1 (1.0%)	
			Phlegm on normal CT chest	1 (1.0%)	
Montcuquet et al.^20^	67	9 (13.4%)	Gastrointestinal side effects	6 (9.0%)	9 (13.4%)
			Infection	3 (4.5%)	
			Deranged liver enzyme	18 (20%)	
			Hyperbilirubinemia	2 (2.2%)	
			Respiratory infection	11 (12.2%)	
			Urinary tract infection	5 (5.6%)	
			Varicella-zoster virus infection	5 (5.6%)	
			Anemia	6 (6.7%)	
			Leukopenia	4 (4.4%)	
			Rectal cancer	1 (1.1%)	
			Renal insufficiency	1 (1.1%)	
			Hair loss	2 (2.2%)	
Huang et al.^25^	90	39 (43%)	Diarrhea	2 (2.2%)	8 (9%)
Jiao et al.^15^	109^a^	21 (19%)	Hair loss	5 (4.6%)	1 (0.9%)
			Mildly elevated liver enzyme	3 (2.8%)	
			Diarrhea and abdominal pain	2 (1.8%)	
			Constipation	1 (0.9%)	
			Leukopenia	3 (2.8%)	
			Thrombocytopenia	1 (0.9%)	
			Shingles	2 (1.8%)	
			Herpes simplex infection	2 (1.8%)	
			Headache	2 (1.8%)	
			Chronic dermopathy of hands and nail	1 (0.9%)	
Mealy et al.^24^	245	NR	NR	NR	NR
Yang et al.^21^	30	3 (10%)	Mildly elevated liver enzyme	2 (6.7%)	0 (0%)
			Nausea	1 (3.3%)	
Zhou et al.^26^	31	NR	NR	NR	NR
Poupart et al. ^22^	42	5 (11.9%)^b^	Serious infection events	5 (11.9%)	5 (11.9%) due to thrombocytopenia, arthromyalgia, Gastrointestinal side effects

*NR* not reported.

^a^Total number of patients = 109 (86 of them received MMF > 6 months and were included in efficacy assessment).

^b^The article did not report adverse events other than serious infection events.

## Discussion

Our analysis, including 15 retrospective studies, showed that treatment with MMF for 13–95 months in NMOSD patients had significantly reduced ARR with a relapse-free rate of approximately 60% (ranged from 27 to 80%) and most of the studies showed EDSS stabilization or improvement varying from 87 to 100%.

After excluding pediatric NMOSD patients from one study^26^, the median age of onset of our study varied from 28.7 to 56.0 years old. For those who underwent the meta-analysis, the post-treatment reduction of ARR decreased approximately 1.13 times a year, and EDSS reduction was 0.85 points, compared to those before treatment initiation in NMOSD patients. The degree of disability measured by EDSS depends mainly on ambulation and EDSS at 3 or 4 is defined as full ambulation. The small change in EDSS at a higher level has a greater disable impact than the same amount of EDSS change at a lower scale. At the pre-treatment state, our analysis composed of 12 studies (9 studies individually and 3 studies in meta-analysis) with a moderately severe disability of median EDSS between 3.0 and 4.0, one^11^ with severe pre-treatment disability with a median EDSS of 6.0, and one study with mild to moderate disabilities with a baseline EDSS of 2.0^19^. Therefore, the reversibility of permanent damages may not be obvious since most of the patients in our analysis could ambulate. Our study also showed that 46–80% of the NMOSD patients were free from relapses. Since disability in NMOSD patients is related to attacks and the accumulation of incomplete recovery, reducing the number of attacks should result in fewer neurological deficits^28^. The findings in our study suggested that MMF exerted positive effects in preventing future relapses and considerably decreasing disability measured by EDSS.

AQP4-IgG autoantibody was present in 84.6% of the total NMOSD patients (range 36.4% to 100%). Two out of the 15 studies^20,24^ evaluated efficacy on MMF treatment separately between seronegative and seropositive NMOSD groups. Although with only 35 seronegative NMOSD, it seemed to show no different in treatment response, with a relapse-free rate around 60% between the two groups; however, no changes in EDSS were demonstrated.

Novel medications such as eculizumab, satralizumab, and inebilizumab have been recently approved for maintenance treatment in NMOSD. Although they showed higher efficacy in relapse reduction, varying from 74–94% in AQP4-positive NMOSD, the benefit was not seen across all of the studies in the AQP4-Ab negative NMOSD group^4–7^. The relapse-free rate is high, around 76.5–97.9% in AQP4-positive NMOSD; however, it varies around 56–84.4% during the 48–96 weeks treatment period for AQP4-Ab negative NMOSD^4–7^. Therefore, the efficacy of the new medications in AQP4-negative groups needs further studies. To date, the data for treatment of seronegative NMOSD patients has no robust evidence. Our study suggests that MMF may be useful in this group of patients.

A large international cohort study revealed that race affected the clinical phenotype, the age at onset, and the severity of attacks^29^. MMF was used as a first-line treatment in 23% for Asians, 13% for Caucasians, and 27% for Afro-America/Afro-European NMOSD patients with a relapse-free rate of approximately 48%; 54% in Asian and 41% in Caucasian. However, the overall outcome is most dependent on early and effective immunosuppressive treatment^29^. Therefore, treatment response in specific groups of patients and further studies on pharmacogenomics are needed to understand the effect of racial difference and response to IS^29^.

A previous study suggested that MMF's efficacy with or without low dose steroids is not statistically different^16^. Adding supraphysiologic doses of steroids may increase MMF's efficacy and increase the risk of infections^30^. Most of the studies in our analysis reported that one- to two-thirds of patients had concomitant use of corticosteroids. Four studies reported concomitant use of MMF with corticosteroid in almost all of the patients^19,21,25,26^. However, details regarding the dose and duration of steroid treatment used in each study were not available. Future study on the benefit or risk of MMF with and without steroids is needed.

The efficacy of MMF was comparable to that of AZA but with fewer ADRs^17–19,21,31,32^. Besides, MMF therapy has been escalated when treatment with AZA showed a suboptimal response, or patients cannot tolerate AZA's side effects^25,27^. Huang et al. compared MMF's efficacy and safety with RTX, AZA, CYP, and cyclosporine A (CyA) and found that MMF was superior to AZA and CYP but inferior to RTX and CyA^33^. However, MMF had the highest tolerability among all IS in the study^33^.

The present study demonstrated that common ADRs were infections, abnormal liver function tests, hair loss, GI symptoms, and bone marrow suppression. ADRs reported from other studies included infections, bone marrow suppression, and malignancy^34^. Increased risk of malignancy has not been proven in another study^35^. ADRs from MMF was not severe nor life-threatening. Only 27 patients (3.9%) discontinued MMF due to ADRs, 3 cases with fatalities, one from infections^20^, and the other two seem to be related to NMOSD^11,25^. These findings are also consistent with other studies reporting tolerable side effects of MMF compared to other IS, e.g., AZA, CYP, or RTX, which led to better drug compliance^33^.

So far, the novel drug reports no serious side effects but particular caution is needed with regards to respiratory tract and urinary tract infection; however, long term side effects require evaluation.

Even though the new medications showed very high efficacy, the medication and accessibility cost hampers the use of the new drug. Furthermore, it is still unclear whether the new drug should be used as a first-line treatment or escalated when patients do not respond to other IS. Currently, MMF is among the first-line maintenance therapy for NMOSD, especially in Asia, where it is widely available for use and at affordable prices.

## Limitation

Our analysis has several limitations. Firstly, the studies included in this review were mostly observational cohort studies subjected to particular bias. Secondly, the study populations' heterogeneity, particularly racial differences, pre-treatment disability, and frequency of relapse, reflected the severity of the disease, various MMF dosage use, and concomitant corticosteroid use before using other IS, which could contribute to different treatment outcomes and ADRs. MMF's efficacy in NMOSD patients should be cautiously interpreted and need further studies; nevertheless, it seems to show reasonable effects for relapse prevention in NMOSD patients.

## Conclusion

This systematic review and meta-analysis indicate that receiving MMF as a preventive therapy in NMOSD patients is associated with a reduction in ARR and EDSS compared to pre-treatment use. It also has acceptable ADRs and low rates of discontinuation.

## Supplementary information

Supplementary Information.

Supplementary Table 1.

## Data Availability

All data generated or analysed during this study are included in this published article.

## Abbreviations

NMOSD: Neuromyelitis optica spectrum disorders
AQP-4 IgG: Aquaporin-4 immunoglobulin G
ON: Optic neuritis
TM: Transverse myelitis
MS: Multiple sclerosis
AZA: Azathioprine
MMF: Mycophenolate mofetil
CYP: Cyclophosphamide
MPA: Mycophenolic acid
IMPDH: Inosine-5′-monophosphate dehydrogenase
ADRs: Adverse drug reactions
ARR: Annualized relapse rate
EDSS: Expanded Disability Status Scale
SD: Standard deviation
MD: Mean difference
IS: Immunosuppressive agents
GI: Gastrointestinal
RTX: Rituximab
CyA: Cyclosporine A
