# Supplementary material for: Efficacy and safety of mycophenolate mofetil therapy in neuromyelitis optica spectrum disorders: a systematic review and meta-analysis
Source: Sci Rep. 2020 Oct 7;10:16727. doi: 10.1038/s41598-020-73882-8 (PMC7541495; doi:10.1038/s41598-020-73882-8)
Supplement: Supplementary file 1 — Supplementary Information. [file 41598_2020_73882_MOESM1_ESM.docx]

**Efficacy and Safety of Mycophenolate Mofetil Therapy in Neuromyelitis Optica Spectrum Disorders: A Systematic Review and Meta-Analysis**

Sakdipat Songwisit^1^, Punchika Kosiyakul^1^, Jiraporn Jitprapaikulsan, MD. ^2,3^, Naraporn Prayoonwiwat, MD.^2,3^, Patompong Ungprasert, MD.^4^, Sasitorn Siritho, MD.^2,3,5^

**Supplementary data: Search terms**

General search and Medical Subject Headings (MeSH) terms were used as follows:

EMBASE

‘Mycophenolate mofetil’ OR Mycophenolate mofetil OR

‘Mycophenolic acid’ OR Mycophenolic acid OR

‘Mycophenolate’

AND

‘Myelooptic neuropathy’ OR Myelooptic neuropathy OR

‘Neuromyelitis optica’ OR

‘Neuromyelitis optica spectrum disorder’ OR

‘NMO’ OR

‘NMOSD’ OR

‘Devic disease’ OR

OVID MEDLINE

‘Mycophenolate mofetil’ OR

‘Mycophenolic acid’ OR

‘Mycophenolate’ OR

AND

‘Myelooptic neuropathy’ OR

‘Neuromyelitis optica’ OR

‘Neuromyelitis optica spectrum disorder’ OR

‘NMO’ OR

‘NMOSD’ OR

‘Devic disease’ OR
