# Supplementary material for: Efficacy and safety of mycophenolate mofetil therapy in neuromyelitis optica spectrum disorders: a systematic review and meta-analysis
Source: Sci Rep. 2020 Oct 7;10:16727. doi: 10.1038/s41598-020-73882-8 (PMC7541495; doi:10.1038/s41598-020-73882-8)
Supplement: Supplementary file 2 — Supplementary Table 1. [file 41598_2020_73882_MOESM2_ESM.docx]

**Efficacy and Safety of Mycophenolate Mofetil Therapy in Neuromyelitis Optica Spectrum Disorders: A Systematic Review and Meta-Analysis**

Sakdipat Songwisit^1^, Punchika Kosiyakul^1^, Jiraporn Jitprapaikulsan, MD. ^2,3^, Naraporn Prayoonwiwat, MD.^2,3^, Patompong Ungprasert, MD.^4^, Sasitorn Siritho, MD.^2,3,5^

**Supplementary Table:** Additional details on year and country of publication, total number of patients, recruitment/population of patient

| **Author** | **Year** | **Country** | **Total** | **Recruitment of patients** |
| --- | --- | --- | --- | --- |
| Jacob et al. | 2009 | USA | 24 | Mayo Clinic (Rochester, Minnesota; Scottsdale, Arizona; Jacksonville, Florida) from June 1999 until June 2006 |
| Huh et al. | 2014 | Korea | 58 | National Cancer Center, Yeungnam University College of Medicine, and Korea University Medical Center from March 1, 2009 to March 31, 2013 |
| Mealy et al. | 2014 | USA | 28 | Johns Hopkins University and Mayo clinic |
| Torres et al. | 2015 | USA | 11 | Hospital of the University of Pennsylvania |
| Chen et al. | 2016 | China | 62 | West China Hospital of Sichuan University from January 1, 2010 to January 15, 2015 |
| Jeong et al. | 2016 | Korea | 34 | Research Institute and Hospital of National Cancer Center from May 2005 to July 2014. |
| Xu et al. | 2016 | China | 38 | Department of Neurology Peking Union Medical College Hospital MSNMOBase (since 2011) |
| Chen et al. | 2017 | China | 105 | West China Hospital of Sichuan University and Third Affiliated Hospital of Sun-Yat-Sen University from September 2009 to February 2016 |
| Montcuquet et al. | 2017 | France | 67 | NOMADMUS cohort of NMO, France (since 2011) |
| Huang et al. | 2018 | China | 90 | Third Affiliated Hospital of Sun-Yat-Sen University |
| Jiao et al. | 2018 | China | 86 | China-Japan Friendship hospital from January 2009 to October 2016 |
| Mealy et al. | 2018 | USA | 245 | Johns Hopkins University School of Medicine (Baltimore, MD, USA),  Research Institute and Hospital of National Cancer Center (Goyang, Korea),  The University of Texas Southwestern Medical Center (Dallas, TX, USA),  Mayo Clinic (Scottsdale, AZ, USA),  Charité University Medicine (Berlin, Germany),  Neuroclinica (Medellín, Colombia) |
| Yang et al. | 2018 | China | 30 | Shandong Provincial Hospital from December 1, 2012 to May 31, 2016 |
| Yifan et al. | 2019 | China | 127 | Third Affiliated Hospital of Sun-Yat-Sen University and Guangzhou Women and Children’s Medical center from January 2012 to November 2017 |
| Poupart et al. | 2020 | France | 42 | OFSEP and NOMADMUS study cohorts from April 1993 to June 2018 |
